# Supplementary material for: Proteomic characterization of aging-driven changes in the mouse brain by co-expression network analysis
Source: Sci Rep. 2023 Oct 24;13:18191. doi: 10.1038/s41598-023-45570-w (PMC10598061; doi:10.1038/s41598-023-45570-w)
Supplement: Supplementary file 1 — Supplementary Information 1. [file 41598_2023_45570_MOESM1_ESM.pdf]

Supplemental Data

Proteomic characterization of aging-driven changes in the mouse brain by co-expression network analysis

Kazuya Tsumagari[1,2,3,4,*], Yoshiaki Sato[5], Hirofumi Aoyagi[5], Hideyuki Okano[6], Junro Kuromitsu[5,*]

[1]Center for Integrated Medical Research, Keio University School of Medicine, Shinjuku-ku, Tokyo 160-8582, Japan.
[2]Proteome Homeostasis Research Unit, RIKEN Center for Integrative Medical Sciences, Tsurumi-ku, Yokohama, Kanagawa 230-0045, Japan.
[3]Laboratory for Integrative Genomics, Proteome Homeostasis Research Unit, RIKEN Center for Integrative Medical Sciences, Tsurumi-ku, Yokohama, Kanagawa 230-0045, Japan.
[4]Laboratory for Metabolomics, RIKEN Center for Integrative Medical Sciences, Tsurumi-ku, Yokohama, Kanagawa 230-0045, Japan.
[5]Eisai-Keio Innovation Laboratory for Dementia, Human Biology Integration Foundation, Eisai Co., Ltd., Shinjuku-ku, Tokyo 160-8582, Japan.
[6]Department of Physiology, Keio University School of Medicine, Shinjuku-ku, Tokyo 160-8582, Japan.

***Correspondence**:
kazuya.tsumagari@riken.jp (KT), j-kuromitsu@hhc.eisai.co.jp (JK)
Tel: +81-3-5843-7081 / Fax: +81-3-5315-4534

Contents

Supplementary Figures (.pdf)
- Figure S1. Volcano plots comparing protein expression at 3 months old and 15 months old, and at 15 months old and 24 months old.
- Figure S2. Levels of module eigenproteins.
- Figure S3. Interactome of M1 synaptic module proteins.

Supplementary Tables 1-7 (.xlsx)
- Table S1. TMT channels of samples.
- Table S2. List of quantified proteins in cortex.
- Table S3. List of quantified proteins in hippocampus.
- Table S4. Summary of the number of significantly regulated proteins.
- Table S5. GO term enrichment analysis of significantly regulated proteins.
- Table S6. Result of WGCNA.
- Table S7. GO term enrichment analysis of detected modules.

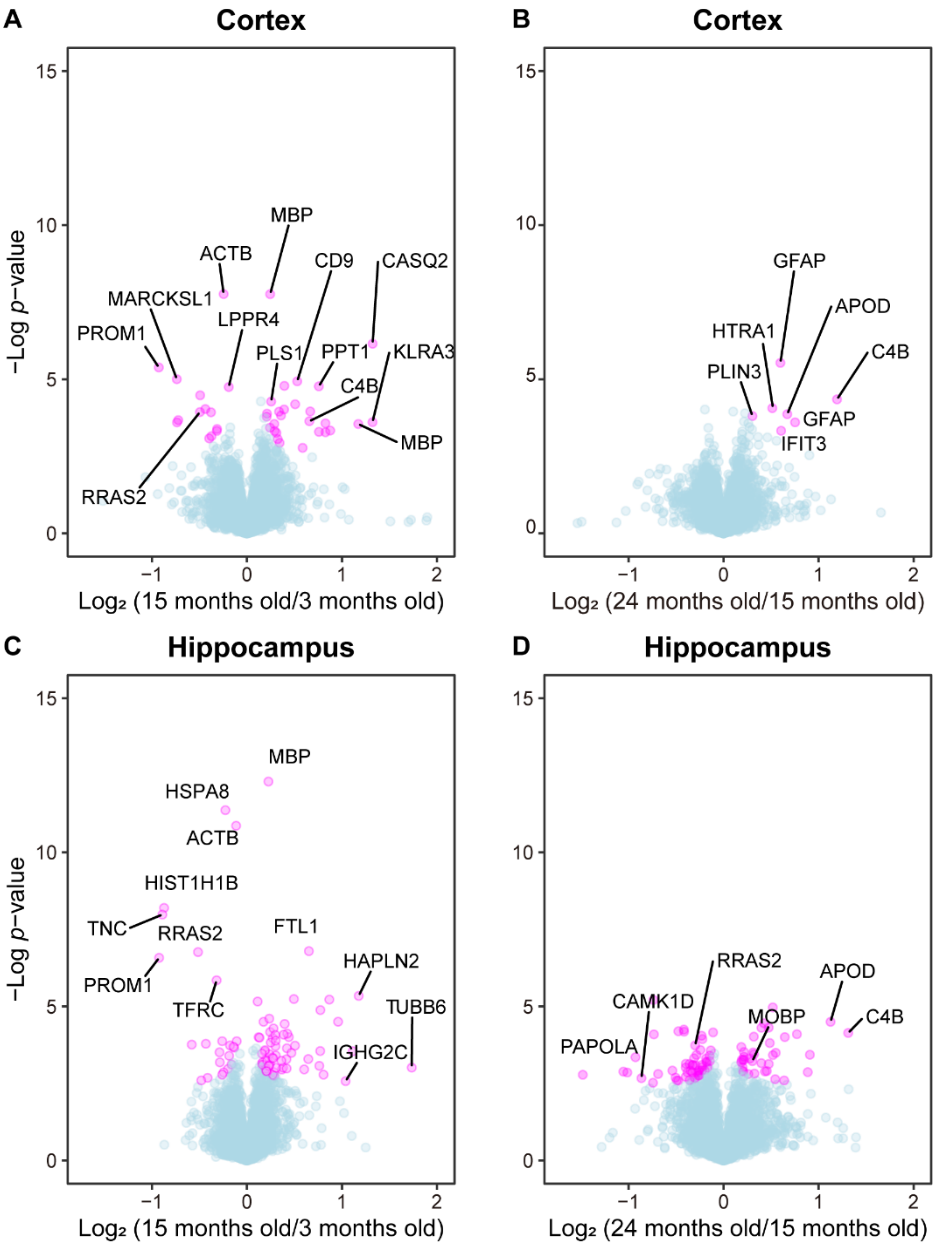

Figure S1. Volcano plots comparing protein expression at 3 months old and 15 months old, and at 15 months old and 24 months old.

Volcano plots comparing protein expression at 3 months old and 15 months old (A and C), and at 15 months old and 24 months old (B and D) in cortex (top row) and hippocampus (bottom row). Welch's t-tests were performed to identify significantly changed proteins (N = 6). The proteins with q-value <0.05 are highlighted with color.

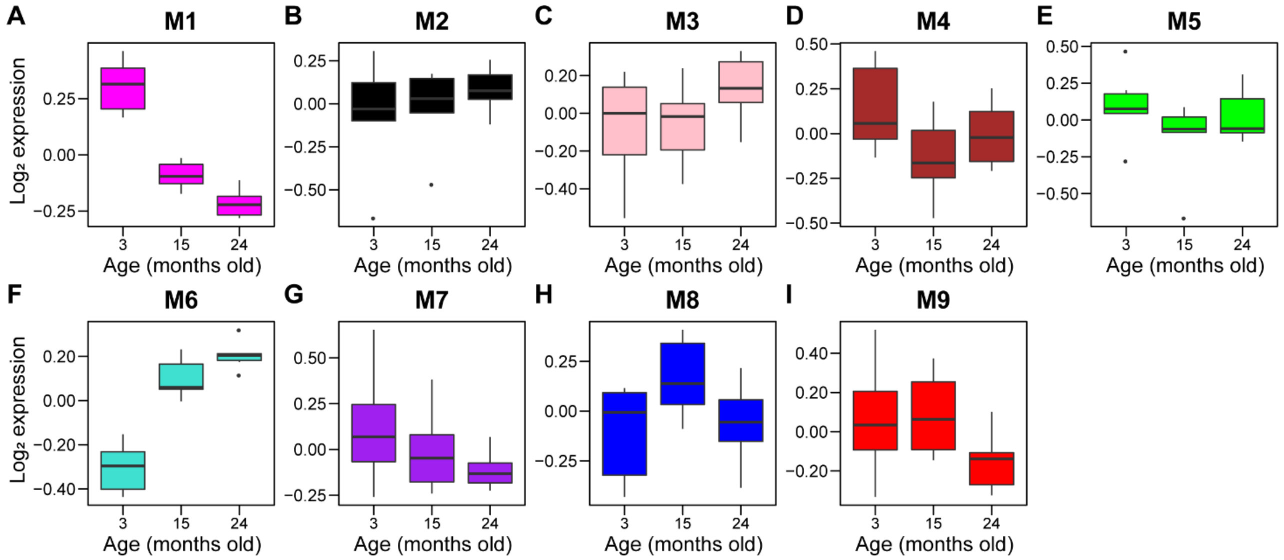

Figure S2. Levels of module eigenproteins.

Module eigenprotein is defined as the first principal component of a given module and serves as a representative of the module.

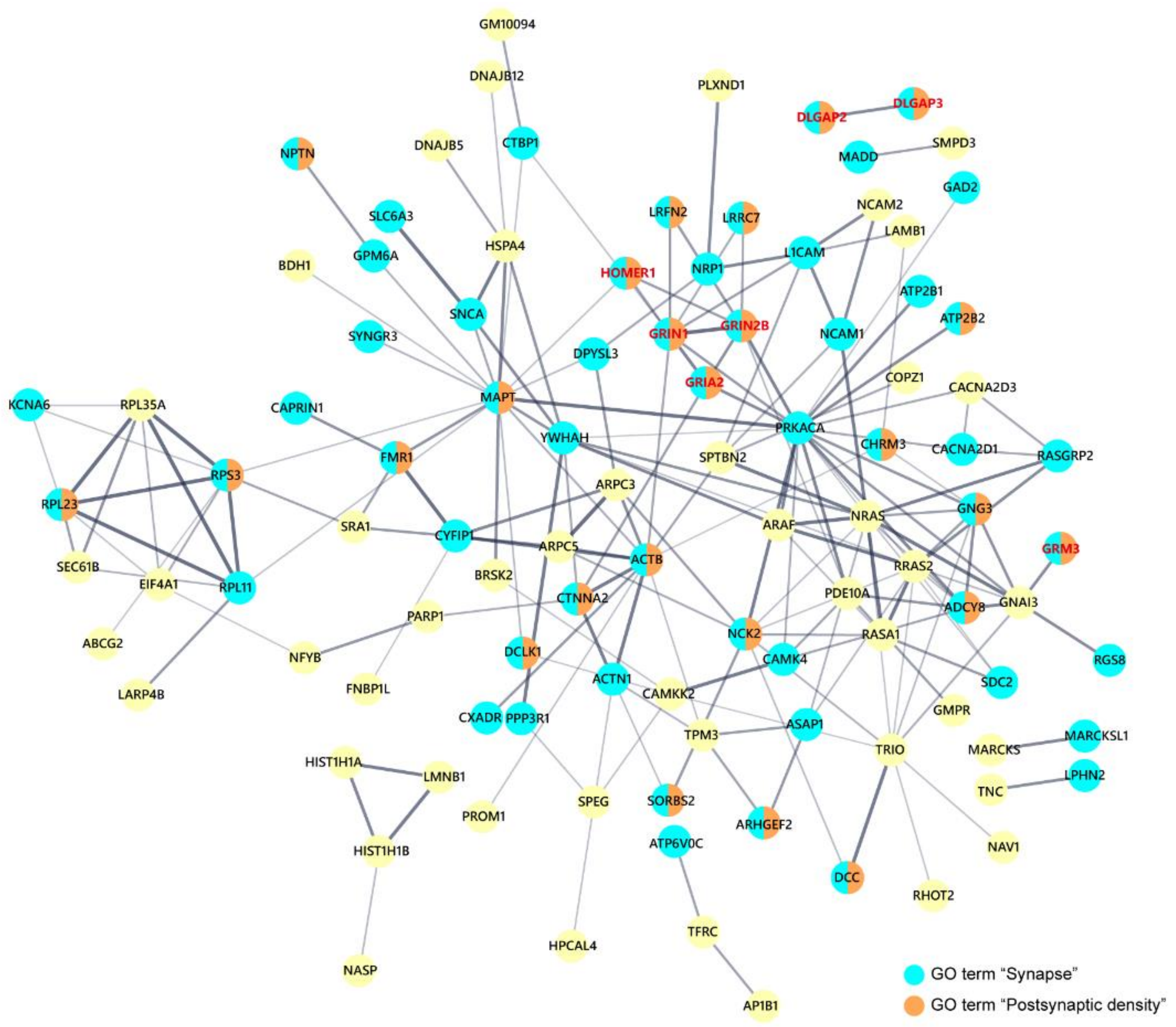

Figure S3. Interactome of M1 synaptic module proteins.

Protein-protein interaction of M1 synaptic module proteins. The proteins with GO terms “synapse” and “postsynaptic density” are highlighted in blue and brown, respectively.
